# Chondroid syringoma on the nasal wing: A case report in a 22-year-old male

**DOI:** 10.1016/j.ijscr.2024.110618

**Published:** 2024-11-19

**Authors:** Bakri Roumi Jamal, Lana Sabbagh, Aya Asfari, Julie Khayat, Alaa Alzakri, Silva Ishkhanian

**Affiliations:** a Faculty of Medicine, University of Aleppo, Aleppo, Syria; b Department of Dermatology, Faculty of Medicine, University of Aleppo, Aleppo, Syria

**Keywords:** Chondroid syringoma, Mixed tumor, Nodule, Benign, Biopsy, Case report

## Abstract

**Introduction and importance:**

Chondroid syringoma also referred to as a mixed tumor, is a benign and rare tumor originating from apocrine or eccrine sweat glands; it predominantly affects middle-aged men. While fine-needle aspiration cytology can aid in diagnosis, histological confirmation remains essential due to the potential for misdiagnosis.

**Case presentation:**

A-22-year-old male who presented with a solitary, slow-growing, painless, erythematous nodule on the right nasal wing that had been present for 1 year, which was misdiagnosed as an epidermal cyst. an excisional biopsy was performed and showed in histological examination foci of myxoid to cartilaginous lakes associated with glandular structures.

**Clinical discussion:**

Chondroid syringoma characterized by mesenchymal and epithelial components. Typically presenting as a painless nodule on the head and neck, CS can be misdiagnosed due to overlapping features with other lesions. Diagnosis involves fine-needle aspiration cytology and histological confirmation. The primary treatment is surgical excision.

**Conclusion:**

Chondroid syringoma has distinctive histological characteristics, which include myxoid to cartilaginous components alongside glandular structures, these features are essential for accurate differentiation from other neoplasms. Surgical excision with an adequate margin of normal tissue is the recommended treatment approach to ensure complete removal and minimize recurrence.

## Highlights

• Chondroid syringoma is a rare skin tumor, affecting 0.01% to 0.098% of cases, predominantly in middle-aged males.
• The tumor typically appears as a firm, painless nodule (1 -3 cm) on the head and neck, including the nose, cheeks.
• Diagnosis often depends on histopathology, as many cases are misidentified as cysts or other lesions, highlighting biopsy's role.
• Histological examination reveals myxoid to cartilaginous lakes and glandular structures, key for distinguishing chondroid syringoma.
• Surgical excision with a margin of normal tissue is the recommended treatment, ensuring complete removal and reducing recurrence risk.

## 1 Introduction

Chondroid syringoma, also referred to as a mixed skin tumor composed of mesenchymal and epithelial cells, is an adnexal neoplasm originating from either apocrine or eccrine sweat glands [1]. This benign and rare tumor has an incidence ranging from 0.01 % to 0.098 % of all primary skin neoplasms. It predominantly affects males, particularly middle-aged men [2,3].

Chondroid syringoma presents as a firm dermal or subcutaneous nodule that gradually enlarges, averaging 1 to 3 cm in size. It is usually solitary, painless, and asymptomatic, and is typically found on the head and neck, including the lips, nose, cheeks, and scalp [[2], [3], [4], [5]].

Accurate diagnosis of this tumor relies on histopathology. Many cases are initially misdiagnosed as cysts, dermal nevi, or other cutaneous adnexal neoplasms and are often correctly identified only after biopsy analysis [3,6].

This manuscript was prepared by the SCARE 2023 guidelines. [7]

## 2 Case presentation

**Fig. 1:**
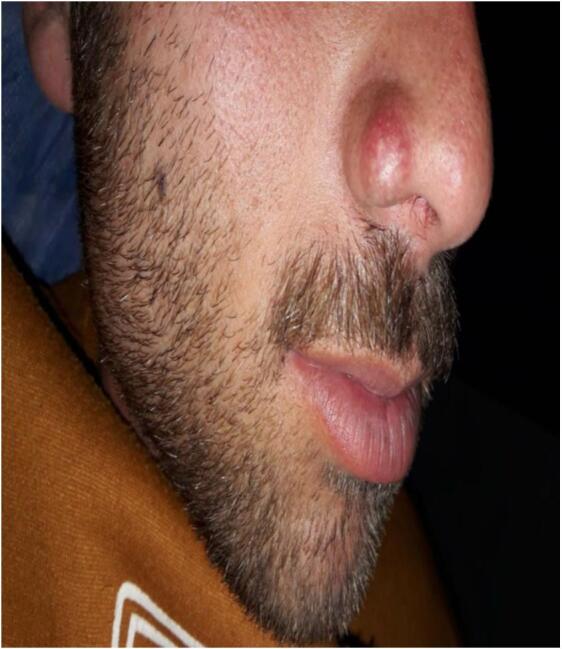
Chondroid syringoma nodule on the nasal wing.

A 22-year-old male presented with a solitary, slow-growing, painless, erythematous nodule on the right nasal wing that had been present for 1 year (Fig. 1). On examination, the swelling was nodular, firm, non-tender, and had surface irregularities, measuring 1.4 × 0.9 × 0.6 cm.

**Fig. 2:**
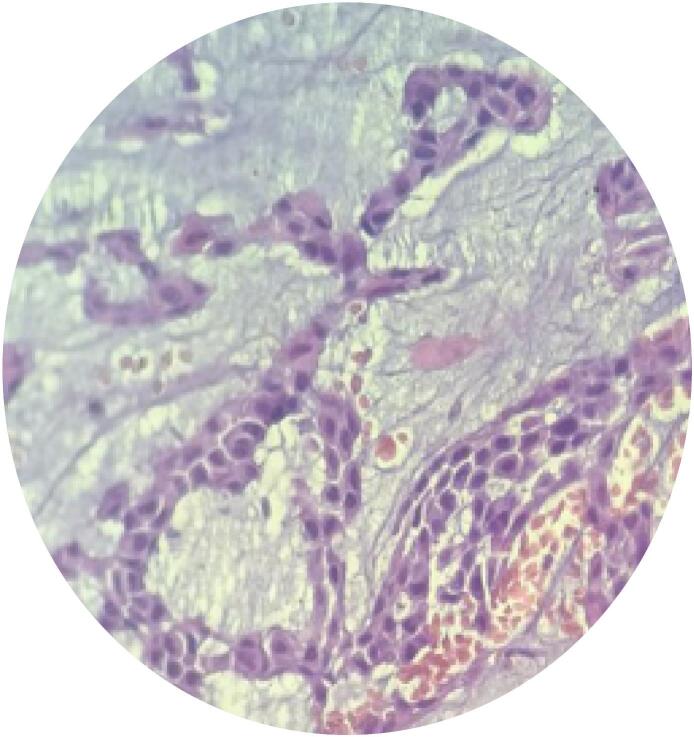
Histologic picture shows epithelial and myoepithelial in chondroid stroma and areas of hyalinization.

**Fig. 3:**
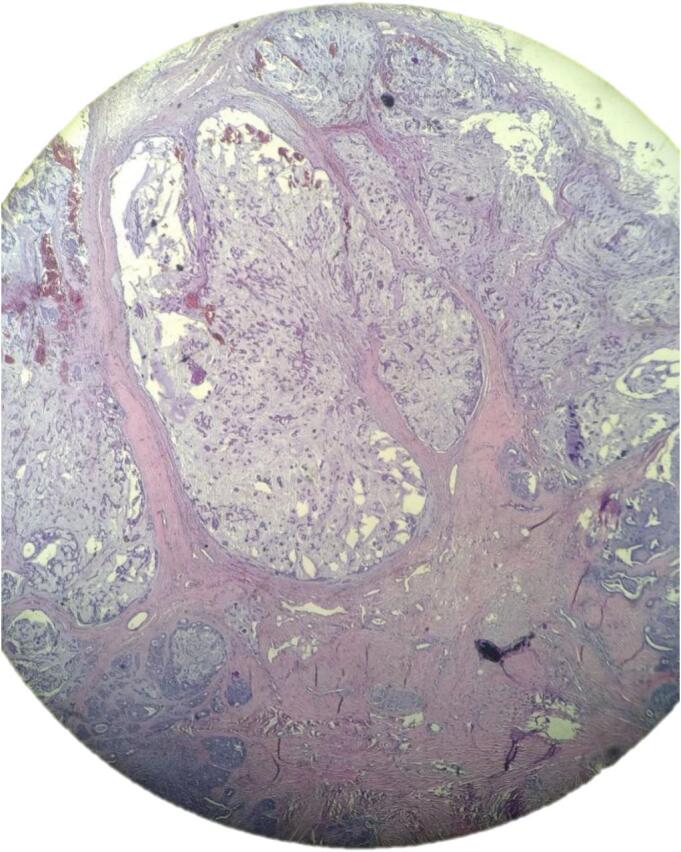
Histologic picture shows apocrine glands were seen with few eccrine glands.

An intralesional triamcinolone acetonied, but it did not yield any results. Subsequently, an excisional biopsy was performed. Histological examination revealed sections showing foci of myxoid to cartilaginous lakes associated with glandular structures. Additionally, keratin-filled cysts were present, and the stroma showed areas of hyalinization (Fig. 2, Fig. 3).

The patient has undergone follow-up for 6 months without any complications or adverse effects appearing.

## 3 Discussion

Chondroid syringoma (CS), also known as mixed tumor of the skin, is a benign and rare tumor with an incidence ranging from 0.01 % to 0.098 %, containing both mesenchymal and epithelial components [6]. First reported by Billroth in 1859 [1,3], the term “Chondroid Syringoma” was officially confirmed by Hirsh and Helwing in 1961 [3], due to the presence of sweat gland components within a cartilaginous stroma [1].

(CS) typically occurs in individuals aged 20–40 years, with male predominance [6]. Clinically, it presents as a painless, firm, slow-growing, red or skin-colored nodule with a smooth surface, ranging in size from 0.5 to 3 cm [4,6].

(CS) is usually found on the head and neck region, including the nose, cheek, upper lip, forehead, and chin [3,4]. It is less common on the hand, foot, penis, scrotum, eyelids, torso, and nasomaxillary groove [3,4]. This tumor shares some clinical and histological features with pleomorphic adenomas, as both present as painless, slow-growing nodules. However, pleomorphic adenomas originate from the salivary or lacrimal glands, whereas (CS) arises from the sweat glands [1].

The differential diagnosis for CS includes basal cell carcinoma, sebaceous cyst, adnexal tumor, and dermatofibroma [8]. In our case, the initial diagnosis was an epidermal cyst. Fine-needle aspiration cytology (FNAC) is commonly used for diagnosing dermal lesions due to its minimally invasive, easy, and cost-effective nature [8]. Diagnosing (CS) requires identifying both plasmacytoid epithelial and myoepithelial cells alongside a fibrillary chondromyxoid matrix [8]. However, (FNAC) has a limitation; aspiration must be taken from multiple areas of the lesion to avoid a higher probability of misdiagnosis [8]. Histological confirmation remains crucial. According to Hirsh and Helwing, the biopsy should exhibit ducts containing one or two rows of cuboidal cells, keratinous cysts, groups of cuboidal or polygonal cells, intercommunicating tubulalveolar complexes lined with two or more rows of cuboidal cells, and a matrix [6].

Genetically, recent studies have shown that (CS) is associated with fusion genes (NDRG1-PLAG1) and (TRPS1-PLAG1), as well as rearrangements of PLAG1 [1]. The recommended treatment for benign (CS) is surgical excision with a margin of normal tissue, preserving functional or aesthetic structures. Other treatment options include dermabrasion, electrodessication, and vaporization with (CO2) laser or argon [3]. Follow-up is advised due to the potential for recurrence [9] or malignant transformation [3].

Malignant (CS) is extremely rare, usually larger than 3 cm, and more often occurs in younger individuals with a female predominance [3]. Histological examination of malignant (CS) shows tumor necrosis, invasive borders, cytologic atypia, and metastases to bone, internal organs, and lymph nodes [3]. Treatment involves aggressive surgery followed by adjuvant radiotherapy, with or without chemotherapy, and regular follow-up to prevent recurrence [3].

## 4 Conclusion

To conclude, chondroid syringoma, is a rare and benign growth. It clinically manifests as a firm, painless nodule ranging from 0.5 to 3 cm in size, typically located on the head and neck. The tumor's unique histological features, including myxoid to cartilaginous components and glandular structures, are critical for differentiation from other neoplasms. The recommended treatment is surgical excision with a margin of normal tissue.

## Abbreviations

CS: Chondroid Syringoma
FNAC: Fine-needle aspiration cytology

## Ethical approval

Not required for case reports at our hospital. Single case reports are exempt from ethical approval in our institution.

## Source of funding

There are no funding sources.

## Consent

Written informed consent was obtained from the patient for publication and any accompanying images. A copy of the written consent is available for review by the Editor-in-Chief of this journal on request.

## CRediT authorship contribution statement

Silva Ishkhanian supervised the manuscript. Alaa Alzakri diagnosed the case and performed the surgery.

Bakri Roumi Jamal and Lana Sabbagh and Aya Asfari and Julie Khayat wrote the manuscript. Bakri Roumi Jamal critically revised the manuscript. All authors read and approval the final manuscript.

## Declaration of competing interest

There are none to declare.

## Data Availability

All data generated or analyzed during this study are included in this published article and its supplementary information files.
